# Strong time dependence of ocean acidification mitigation by atmospheric carbon dioxide removal

**DOI:** 10.1038/s41467-019-13586-4

**Published:** 2019-12-06

**Authors:** M. Hofmann, S. Mathesius, E. Kriegler, D. P. van Vuuren, H. J. Schellnhuber

**Affiliations:** 1 Potsdam Institute for Climate Impact Research, Potsdam, Germany; 2 GEOMAR Helmholtz Centre for Ocean Research Kiel, Kiel, Germany; 3 Copernicus Institute for Sustainable Development, University Utrecht, Utrecht, Netherlands; 4 PBL Netherlands Environmental Assessment Agency, The Hague, Netherlands

**Keywords:** Ocean sciences, Marine chemistry

## Abstract

In Paris in 2015, the global community agreed to limit global warming to well below 2 ${}^{\circ }$C, aiming at even 1.5 ${}^{\circ }$C. It is still uncertain whether these targets are sufficient to preserve marine ecosystems and prevent a severe alteration of marine biogeochemical cycles. Here, we show that stringent mitigation strategies consistent with the 1.5 ${}^{\circ }$C scenario could, indeed, provoke a critical difference for the ocean’s carbon cycle and calcium carbonate saturation states. Favorable conditions for calcifying organisms like tropical corals and polar pteropods, both of major importance for large ecosystems, can only be maintained if CO${}_{2}$ emissions fall rapidly between 2025 and 2050, potentially requiring an early deployment of CO${}_{2}$ removal techniques in addition to drastic emissions reduction. Furthermore, this outcome can only be achieved if the terrestrial biosphere remains a carbon sink during the entire 21st century.

## Abstract (web summary)

Carbon dioxide removal technologies are often touted as a potential strategy to combat ocean acidification. However, the authors show here that these strategies are only effective when included as part of aggressive and rapid climate-action, undermining the idea of geoengineering as a panacea.

## Introduction

Unrestrained anthropogenic carbon dioxide (CO${}_{2}$) emissions would not only cause global mean surface temperatures to exceed the Holocene range^1^ but also modify the ocean chemistry in an unprecedented way^2^. The Paris Agreement, struck at the 21st Conference of the Parties (COP21) in Paris in 2015, intends to limit global warming to 1.5–2 ${}^{\circ }$C. This multilateral consensus is based on the assessment that highly disruptive environmental impacts could be expected if these targets are exceeded^3–5^. Research shows that reaching the 2 ${}^{\circ }$C goal with reasonable probability already requires ambitious mitigation efforts worldwide^6^. However, while the 2 ${}^{\circ }$C target is assumed to be sufficient to prevent reaching most of the climate system’s tipping points^3,5^, it might not be enough to keep the oceans biogeochemistry and ecosystems intact^7,8^.

This is of particular concern, because once the ocean is severely altered by warming and acidification, it would take many centuries to bring it back to the preindustrial state^7^, even long after the atmospheric CO${}_{2}$ concentration has returned to its preindustrial level. This slow response of the ocean to atmospheric changes is in part related to the long time scale of the overturning circulation, where water masses can be out of contact with the atmosphere for more than 1000 years before they are completely circulated back to the surface and re-establish an equilibrium with atmospheric CO${}_{2}$ concentrations and temperatures.

Approximately 26% of current anthropogenic CO$${}_{2}$$ emissions have been absorbed by the oceans already^9^, which has reduced the oceans $$p$$H value from 8.21 to 8.10 (ref. ^1^). This trend is a serious threat to many marine organisms, especially calcifying species that require seawater with an aragonite saturation state larger than one ($${\Omega }_{{\rm{a}}}$$ $$> $$ 1) to build shells and skeletons. Among the most important calcifiers are tropical reef-building corals and pteropods, planktonic snails dwelling in the pelagic zone. Both are known to be threatened by global warming and ocean acidification^10–13^. Coral reefs are among the most important ecosystems because they provide habitat to more than a million species and ecosystem services to more than hundreds of millions of people^14^. As a result of marine heatwaves, overfishing, pollution, storms and unsustainable coastal development, the distribution and abundance of tropical corals has been reduced by approximately 50% over the past 30 years^5^. Marine heatwaves lead to coral bleaching and become more frequent with global warming. Numerous studies have shown that even a limitation of global warming to 2 $${}^{\circ }$$C compared to preindustrial conditions will put almost all tropical coral reefs at risk^11,12,15^. Ocean acidification puts additional pressure on corals because it reduces the saturation state of aragonite, with the result that corals have to spend more energy on calcification, grow slower, get more vulnerable to diseases and become less competitive^5,16^. The weakening of coral reef resilience can have the consequence that macroalgae overgrow the corals to the extent that the whole reef shifts to an algal-dominated regime^10^ with reduced biodiversity. The other calcifiers addressed here, pteropods, are small planktonic molluscs that produce thin aragonite shells and therefore require an environment that is oversaturated with respect to aragonite ($${\Omega }_{{\rm{a}}}\ > \ 1$$)^17^. They are highly abundant in temperate and polar waters^5^ and play a crucial role in the marine food web^13^, because they provide a link between phytoplankton and fish, birds and whales^5^. The reduction in the aragonite saturation state $${\Omega }_{{\rm{a}}}$$ already affects the ability of pteropods to produce shells, swim and survive^5,18^. Especially at high latitudes, where ocean acidification is most severe, large regions are expected to become uninhabitable for pteropods, as we show below.

Advocates of planetary-scale interventions have argued that artificial carbon dioxide removal (CDR) from the atmosphere might solve both global warming and acidification problems. However, it has been shown^7^ that even extreme CDR interventions would fail to restore marine chemistry within many centuries, if they are deployed too late, i.e., after the ocean has already been severely altered^7,19,20^. This insight is critical for the scientific assessment of the purported benefits of geoengineering measures^21–25^. It has been maintained^23,26^ that planetary-scale technical remedies offer an ultimate chance of saving the global environment once mitigation policies become insufficient and that CDR can be an effective response because it reduces both climate and acidification risks^26^. However, CDR can only be effective if deployed early and combined with massive mitigation strategies, which has been found in several studies on 2 ${}^{\circ }$C pathways that combine stringent mitigation strategies and CDR^27–29^. The amount of CO${}_{2}$ that can be removed from the atmosphere by CDR options is limited by land availability, storage capacity, water requirements or energy consumption^30^, thus cannot be expected to counteract unmitigated emissions.

Here, we show that deep mitigation pathways, such as Shared Socioeconomic Pathway 1 (SSP1)-2.6, may also limit ocean acidification when embedded into aggressive climate-action scenarios^28,29^. This study aims to demonstrate the importance of correct timing for CDR deployments as an accompanying measure for existing and ambitious CO${}_{2}$ emission reduction pathways to maintain global warming within the safe range and efficiently protect the marine environment. More specific, we investigate whether the limited and timely application of atmospheric CDR could help to maintain global warming in the range between 1.5 and 2 ${}^{\circ }$C and simultaneously prevent calcifying organisms, such as tropical coral reefs and pteropods, from serious damages. In previous studies by Vaughan et al.^24^, Frölicher and Joos^20^ and Mathesius et al.^7^, the importance of the timing of mitigation and CDR measures, with respect to the irreversibility of ocean acidification and their potential impacts on marine biota, was already highlighted. Based on the findings of these previous studies, our work tries to answer the following questions:

Given that society would decide on the deployment of atmospheric CDR starting as early as possible (i.e., during the mid 2020s), would it matter if we postponed this deployment by 50 years under the constraint of removing exactly the same amount of CO${}_{2}$ as in a case with earlier action? Would the benefits of delayed CDR measures be comparable to those of early CDR deployment? The outcome of our research suggests that only an early deployment of CDR would have the potential to mitigate damages on marine ecosystems due to ocean acidification, projected to occur even under the low emission SSP1-2.6 scenario, by concomitantly limiting global warming to 1.5 ${}^{\circ }$C. In this context, CDR only makes sense if rapid decarbonization of the world economy succeeds—not if it founders.

## Results

### Model experiments and strategy

**Fig. 1:**
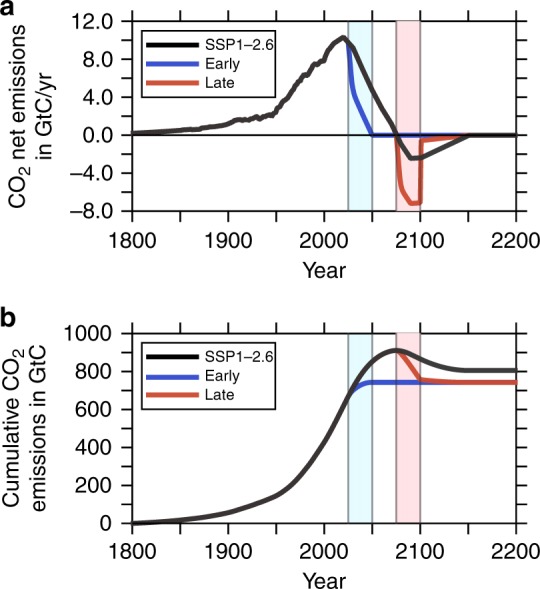
Emission scenarios utilized in this study. Time line of the three anthropogenic CO${}_{2}$ emission scenarios between 1800 and 2200. **a** CO${}_{2}$ net emissions in GtC year${}^{-1}$. **b** Cumulative CO${}_{2}$ emissions in GtC.

We employed the model CLIMBER-3$\alpha$ + C (see Methods), an Earth system model of intermediate complexity (EMIC) based on the abiotic version of CLIMBER-3$\alpha$^31^, to simulate the following CO${}_{2}$ emissions scenarios from 1800 to 2200:

The baseline scenario SSP1-2.6 (ref. ^27^) was designed to meet the 2 ${}^{\circ}$C climate target. Since the original SSP1-2.6 scenario ends already in 2100, we have extended it until 2200 by linearly reducing the net negative emissions to zero from 2100 to 2150 (see black line in Fig. 1). Compared to the baseline, our first newly designed CDR scenario assumes an additional removal of atmospheric CO${}_{2}$ of in total 109 Gigatons of carbon (GtC) by CDR measures between 2025 and 2050. In 2050, the net emissions reach zero (see blue line in Fig. 1). In a subsequent consolidation phase, CDR guarantees net zero emissions until 2050–2075, by an additional extraction of 64 GtC. In contrast to SSP1-2.6, this modified scenario ignores negative emissions between 2075 and 2200. Henceforth, we refer to this scenario, which extracts a total of 63 GtC more than SSP1-2.6 over the entire CDR period (between 2025 and 2150), as “EARLY”.

The second modification to the baseline scenario was motivated by the question of whether it would make a large difference in the resulting climate and marine ecosystem response if we postpone the CDR interventions suggested by EARLY by 50 years. Here, we initialize the CDR deployment again by removing an extra 109 GtC total between 2075 and 2100 (see red line in Fig. 1) by subsequently decreasing the negative CO${}_{2}$ emissions between 2100 and 2150 to zero, such that the cumulative reduction in comparison to SSP1-2.6 amounts to exactly 63 GtC (equal to the total extra reduction assumed in EARLY). Henceforth, because the CDR starts 50 years later, we refer to this scenario as “LATE”.

In contrast to a similar study by Frölicher and Joos^20^, who demonstrated what would have happened if society had stopped anthropogenic emissions in 2000 in comparison to a weak and strong mitigation scenario until 2100, our work tries to find realistic timing for CDR interventions that are not only eligible to meet the 1.5 ${}^{\circ}$C target but also to protect marine biota.

CLIMBER-3$\alpha$ + C includes an isogeochemical (i.e., it does not account for river-runoff and sedimentation loss) marine carbon cycle model coupled with an NPZD (nutrient, phytoplankton, zooplankton and detritus) biogeochemical model but does not include a state of the art terrestrial carbon cycle and land biosphere model. To emulate the impacts of the terrestrial biosphere on the global carbon cycle, which is an important component of the climate system^32^, CLIMBER-3$\alpha$ + C was coupled with a box model similar to a reduced version of the terrestrial model by Wigley^33^ (see Methods). The three terrestrial boxes accounting for the carbon budgets of living plants, detritus and soil were interactively coupled to the CLIMBER-3$\alpha$ + C atmospheric module. The effects of land use change were not accounted for because the emission scenarios already included them.

The terrestrial box model mainly parameterizes the gross primary production rate (${G}_{P}$) of living plants, respiration rate ($R$) and remineralization and oxidation rates of detritus and soil (${Q}_{10}$ values). The model sustains a net carbon sink over the entire simulation period^34^.

To achieve a quasi-steady state representing the unperturbed preindustrial climate, a spin-up of CLIMBER-3$\alpha$ + C of more than 6000 years was performed. The control model simulation arrives at a climate state comparable to that of the observational data^1^, with an atmospheric CO${}_{2}$ concentration of 284.0 ppmv, and a globally averaged annual mean sea surface pH value of ~8.13.

We performed the first scenario analyses by running the EMIC from 1800 until 2005 with historical CO${}_{2}$ emission data^35^. Subsequently, the model was forced with CO${}_{2}$ emissions by the SSP1-2.6, EARLY, and LATE protocols until the year 2200^29^.

### Emission scenarios

**Fig. 2:**
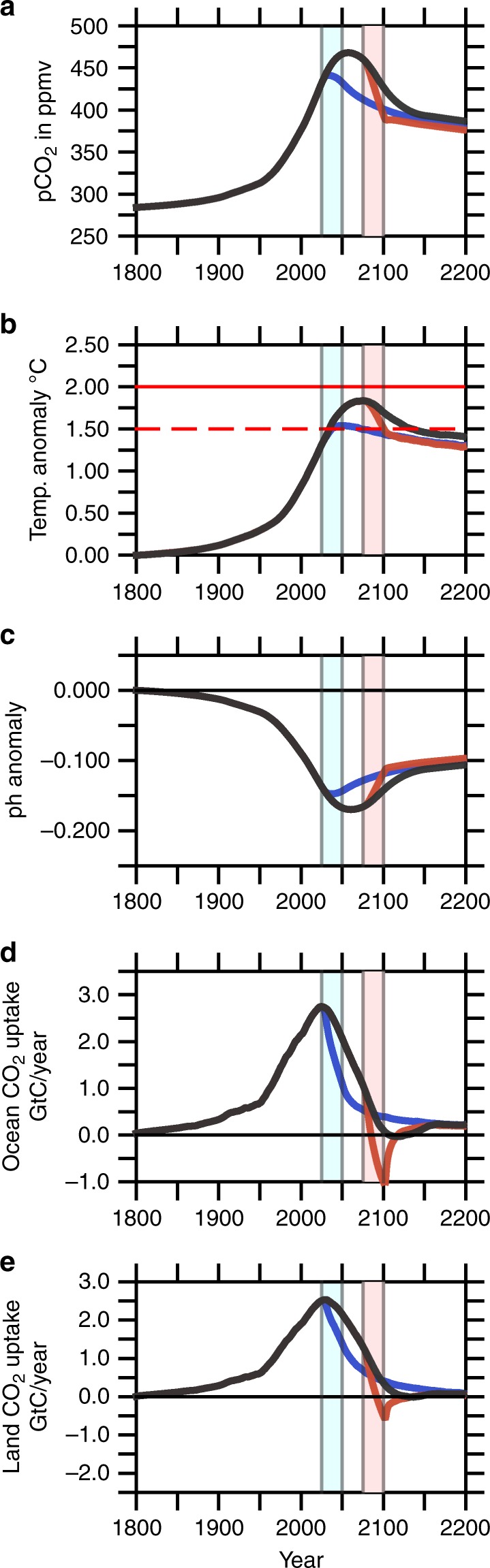
Evolution of important climatological variables. Time evolution of atmospheric and global mean sea surface values of climatologically relevant parameters during the entire model simulation between years 1800 and 2200. **a** atmospheric pCO${}_{2}$ concentrations in ppmv; **b** globally averaged and annual mean atmospheric temperature anomaly at the Earth surface in ${}^{\circ }$C; **c** sea surface pH anomaly; **d** uptake of CO${}_{2}$ by the ocean in GtC year${}^{-1}$; **e** uptake of CO${}_{2}$ by the land biosphere in GtC year${}^{-1}$.The light blue and the pink stripe mark the period of the initial carbon dioxide removal (CDR) measures for the EARLY and LATE scenarios, respectively.

**Fig. 3:**
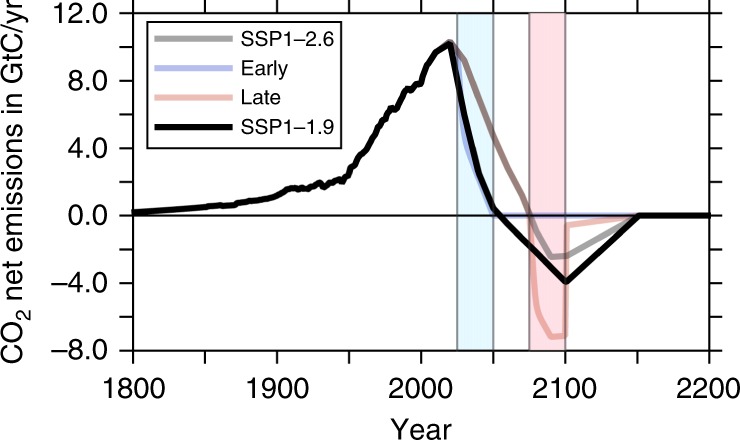
Utilized emission scenarios in comparison to SSP1-1.9. Time line of the three anthropogenic CO${}_{2}$ emission scenarios in comparison with the SSP1-1.9 scenario^38^ between 1800 and 2200 in units of GtC year${}^{-1}$.

In agreement with previous studies^35,36^ forced by SSP1-2.6 emissions, atmospheric levels of CO${}_{2}$ are projected to increase to ~470 ppmv (Fig. 2a), while the global mean temperature peak around year 2075 is expected to be ~1.8 ${}^{\circ}$C above preindustrial values (Fig. 2b) for the SSP1-2.6 simulation. Accordingly, the globally averaged sea surface pH values for the oceans display a pronounced decrease during the middle of the 21st century, exceeding values of −0.17 units (Fig. 2c) for the SSP1-2.6 simulation. Compared to the SSP1-2.6 scenarios over the entire simulation, the scenario EARLY leads to much lower atmospheric CO${}_{2}$ levels, with a maximum of about 440 ppm, resulting in global warming of nearly 1.5 ${}^{\circ }$C and a maximum decrease in pH of approximately 0.15 units, with consecutively decreasing anomalies after the year 2050 (see Fig. 2a–c). The EARLY scenario can be compared with other very low emissions scenarios falling below SSP1-2.6 in the literature. Rockström et al.^37^ proposed a scenario of halving CO${}_{2}$ emissions every 10 years from 2020 onwards that uses CDR only to a very limited extend to reach net zero CO${}_{2}$ emissions by mid century. The lowest SSP scenarios limiting radiative forcing to 1.9 Wm${}^{-2}$ by the end of the century, including an SSP1-1.9 scenario (see Fig. 3) by the IMAGE model, also reach net zero CO${}_{2}$ emissions by mid century^38^. These and similar scenarios were assessed by the recent IPCC Special Report on 1.5 ${}^{\circ }$C (Chapter 2, Rogelj et al., SR1.5)^5^, which found that on average about two thirds of the faster phase out of CO${}_{2}$ emissions compared to associated 2 ${}^{\circ }$C scenarios were due to stronger emissions abatement and only one third due to earlier deployment of CDR. Hence, the acceleration of CO${}_{2}$ phase out compared to SSP1-2.6 does not need to come all from additional CDR as assumed in the EARLY scenario. Obviously, the LATE-scenario is not suited to mitigate climate change efficiently in any case, which notably occurs during the critical time interval between 2025 and 2100.

### CO${}_{2}$ uptake by land and oceans

Continuously rising CO${}_{2}$ emissions have led to elevated atmospheric CO${}_{2}$ levels, which generated a fertilization effect for the land biosphere (Fig. 2e)^33,34^ and increased atmosphere-ocean gradients of the partial pressure of CO${}_{2}$, resulting in a net CO${}_{2}$ flux into the oceans (Fig. 2d)^39^. The average air-to-sea CO${}_{2}$ flux between 1990 and 1999 simulated by SSP1-2.6 amounts to 1.99 GtC per year, while the land biosphere amounts to ~1.85 GtC per year. The numbers agree well with the values given by the Fifth Assessment Report (AR5) of the IPCC^1^ whereby oceans and land take 1.7$\pm$0.5 and 1.4$\pm$0.7 GtC per year during this period of time, respectively. These numbers increase remarkably until 2017 to 2.60 GtC per year for the ocean and 1.54 GtC per year for land in case of SSP1-2.6. During the late twenties in the 21st century, the oceanic uptake of CO${}_{2}$ is projected to reach its maximum at 2.75 GtC per year by SSP1-2.6 (see Fig. 2d) and land will take up a maximum of 2.75 GtC in year 2025. Compared to all other emissions scenarios discussed here, EARLY reveals the lowest cumulative oceanic uptake of atmospheric CO${}_{2}$ between 2025 and 2075, leading to a decrease in global mean sea surface pH of 0.15 units (see Fig. 2c,d).

### Aragonite saturation in polar waters

**Fig. 4:**
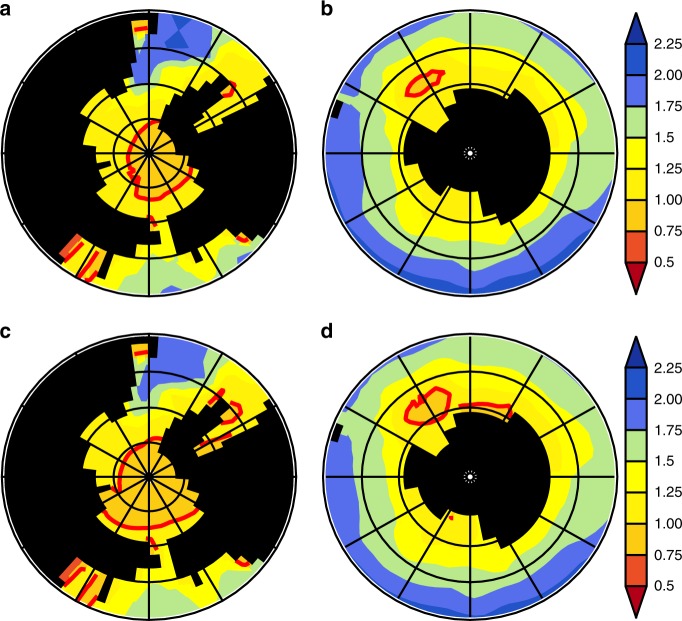
Polar aragonite saturation during boreal winter season. Decadal mean (2070–2080) aragonite saturation state ${\Omega }_{{\rm{a}}}$ during January–March for EARLY **a** Arctic and **b** Antarctic and LATE **c** Arctic and **d** Antarctic. The ${\Omega }_{{\rm{a}}}$ = 1 iso-line is drawn in red.

**Fig. 5:**
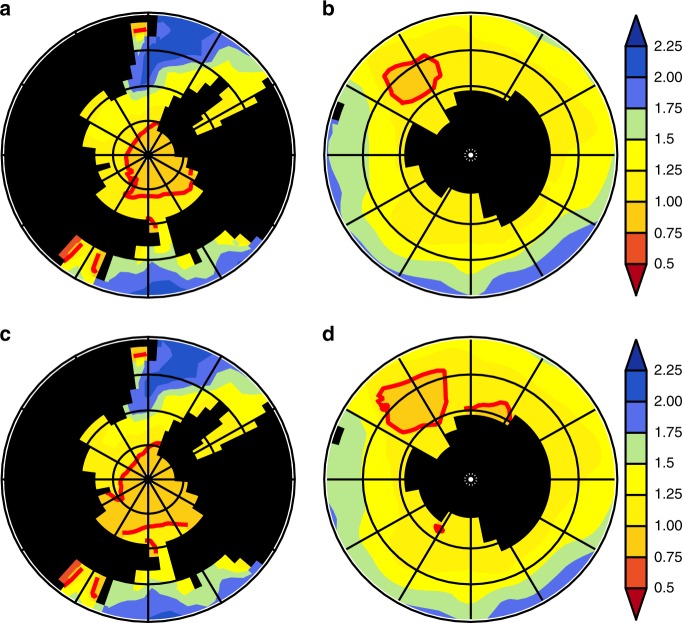
Polar aragonite saturation during boreal summer season. Decadal mean (2070-2080) aragonite saturation state ${\Omega }_{{\rm{a}}}$ during July-September for EARLY **a** Arctic and **b** Antarctic and LATE **c** Arctic and **d** Antarctic. The ${\Omega }_{{\rm{a}}}$ = 1 iso-line is drawn in red.

In line with the findings by Hauri et al.^40^, even in the highly mitigated scenarios (SSP11-2.6), we found a development of a seasonal carbonate undersaturation with respect to aragonite at sea surface. In this regards, our model simulations clearly show the advantage of an early CDR deployment (EARLY) against the LATE-scenario where the action is deferred by 50 years. Figures 4 and 5 show the seasonal mean value of the sea surface aragonite saturation state ${\Omega }_{{\rm{a}}}$ in the Arctic and the Antarctic during the seventies of the 21st century for January until March, and July until September, respectively. In case of EARLY, the maximum area of surface waters during winter season which is undersaturated with respect to aragonite (${\Omega }_{{\rm{a}}}$ $<$ 1) in the Arctic and the Southern Ocean remains below ~5,929,000 km${}^{2}$. For LATE, the winter season area with aragonite undersaturated seawater expanded over the Laptev and Beaufort Seas and most of the Weddell Gyre covers a total of 10,626,000 km${}^{2}$, which is nearly twice the area of the undersaturated water masses in EARLY.

### Coral reef calcification rates

To assess the dependence of gross community calcification in stony corals on sea surface temperature ($T$) and the aragonite saturation state (${\Omega }_{{\rm{a}}}$), we use the empirical rate law by Silverman et al.^41^ which has been derived by utilizing data of >9000 reef locations. Given that ${G}_{{\rm{i}}}$ (see Methods) is the rate of inorganic aragonite precipitation, the gross community calcification rate depends on $T$ and ${\Omega }_{{\rm{a}}}$ as follows:

$${}^{T}{G}_{{\rm{gross}}} \sim {G}_{{\rm{i}}}\cdot exp\left(-{\left(\frac{k^{\prime}_{p} \cdot (T-{T}_{{\rm{opt}}})}{{\Omega }_{{\rm{a}}}^{2}}\right)}^{2}\right),$$

where $k^{\prime}_{p}$ = 1 ${}^{\circ }$C ${}^{-1}$ and ${T}_{{\rm{opt}}}$ represents the optimal temperature for calcification found between 27 and 28 ${}^{\circ }$C^41^.

**Fig. 6:**
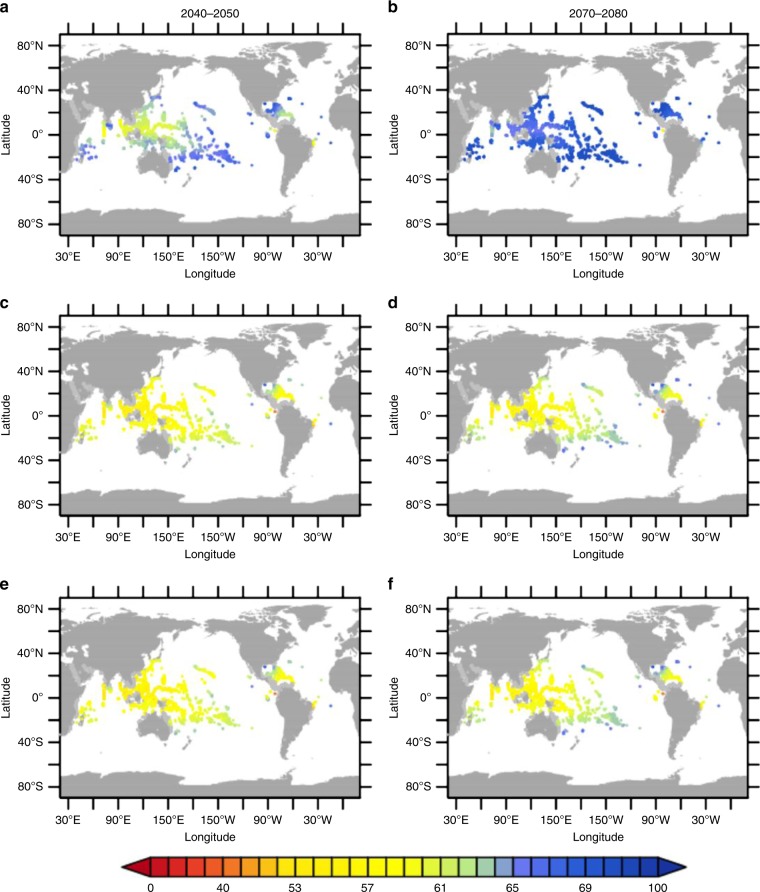
Relative decadal mean gross calcification rates of tropical corals. Global distribution of tropical coral reefs from the Reef Base (M. Tupper et al., ReefBase: A Global Information System on Coral Reefs, http://www.reefbase.org) and their corresponding decadal mean gross calcification rates ${}^{T}{G}_{{\rm{gross}}}$ relative to preindustrial values in %: (2040–2050) **a** EARLY; **c** LATE; **e** SSP1-2.6; (2070–2080) **b** EARLY; **d** LATE; **f** SSP1-2.6.

Here, we apply the relation given above to assess the potential impacts of the three scenarios investigated in our study on the gross community calcification rates of tropical coral reefs during the fourties and the seventies of the 21st century, where the sea surface pH decline is at maximum, by utilizing the summer solstice temperatures (June/December for the northern/southern hemisphere). for ${T}_{{\rm{opt}}}$. Therefore we calculated the change in ${}^{T}{G}_{{\rm{gross}}}$ during these two time intervals relative to their preindustrial values at the more than 10,000 locations in the tropical coral reefs provided by the Reef Base (M. Tupper et al., ReefBase: A Global Information System on Coral Reefs, http://www.reefbase.org). In all scenarios investigated in our study, tropical coral reefs are affected by the impact of anthropogenic CO${}_{2}$ emissions. The largest impacts are projected to occur in the Indonesian archipelago, where the mixed layer depths are relatively shallow, leading to a higher accumulation of anthropogenic CO${}_{2}$ and thus increased ocean acidification. However, there are remarkable differences in the degree of damage among the different scenarios. In the case of EARLY (Fig. 6a), the gross community calcification rate ${}^{T}{G}_{{\rm{gross}}}$ in the Indonesian archipelago during 2040 to 2050 is projected to decline to 60–65% of its preindustrial value, while in LATE (Fig. 6c) and SSP1-2.6 (Fig. 6e), this value only reaches approximately 50–55% of its former strength. Additionally, in the case of EARLY, ${}^{T}{G}_{{\rm{gross}}}$ in the tropical Pacific Ocean and Sargasso Sea declines to 65–70% of its preindustrial value, while in case of LATE and SSP1-2.6, ${}^{T}{G}_{{\rm{gross}}}$ only reaches 60% of its original value. During the seventies, a recognizable advantage of EARLY over LATE is even more pronounced (Fig. 6b, d, f). Coral reefs suffering from reduced biomineralization are at risk to grow malformed sceletons of higher porosity^42^. Although EARLY reveals only a 5–15% improvement of calcification rates over LATE it might be of importance for the success of coral reefs to combat external stressors, such as ocean heat waves leading to bleaching events.

## Discussion

Ocean acidification and climate change, as projected to occur by the mid-21st century, will pose a serious threat to marine biota, even under the most ambitious mitigation strategies (e.g., the SSP1-2.6 emission path). In this study, we focused on the impact of three ambitious scenarios of net CO${}_{2}$ emissons on living conditions for pteropods and tropical reef-building corals. Pteropods play a significant role in the marine foodweb^43^, especially in polar regions. Our study found that large parts in the Arctic and Antarctic are expected to become uninhabitable for pteropods, because severe acidification leads to large areas becoming seasonally undersaturated with respect to aragonite, which is the essential mineral needed for pteropod shells. Previous studies showed that changing water chemistry and temperature already have a negative impact on pteropod survival and shell formation^18,44^. As our model demonstrates, this trend is expected to continue over the next decades, but especially early CDR can prevent large areas in polar regions from becoming undersaturated with respect to aragonite and thus keep those areas habitable for pteropods.

The other important calcifiers highlighted in this study are tropical corals that are the foundation of major biodiversity hotspots in the ocean by providing habitat and resources to over a million reef-associated species^14^. Due to ocean acidification and warming, coral reefs are expected to become severely damaged^12,45,46^. Utilizing an empirical law for the effect of ocean acidification and warming on coral calcification by Silverman et al.^41^, our results suggest that even in the ambitious mitigation scenario SSP1-2.6 the calcification rate of corals will decrease to 50% of the preindustrial level. If, in addition to emissions reduction, CDR is deployed early, the calcification rates will be 5–15 percentage points higher. Reduced calcification rates imply that corals grow slower and have to spend more energy on calcification, which makes it harder for them to compete with macroalgae and seaweeds. This can finally lead to a regime shift from a structurally complex and species-rich reef ecosystem to an algae-dominated ecosystem with lower biodiversity^5,10,16^.

It is important to mention that the future of a coral reef is not only determined by increasing open ocean pH and warming, which is calculated by our model, but also heavily influenced by local factors, such as the reef-specific buffer capacity of seawater, local currents and local overfishing and pollution^47^. To assess future developments of coral reef ecosystems further, regional model studies that can account for local pH variability and extremes are needed, whereas this study demonstrates the overall increasing pressure on coral reefs globally. According to our simulations, early deployment of CDR can contribute to the conservation of coral reefs on a global scale. Although some reefs are more resilient than others^14,47^, it is almost certain that in general the pressure on coral reefs will increase strongly with increasing atmospheric CO${}_{2}$ concentrations, resulting in changing species composition, meaning that vulnerable coral species will be replaced by more resilient corals (e.g., species of the *Porites* genus)^48–50^ or that in severe cases macroalgae will overgrow the whole reef. A global die-back of coral reefs would be accompanied by a loss of the associated ecosystem services that are important for coastal ecosystems like mangrove forests and human societies, e.g., coastal protection, tourism and food security^10^.

In this study, we demonstrated that in combination of reducing CO${}_{2}$ emissions rapidly, early deployment of atmospheric CDR measures could be effective in largely maintaining oceanic physical and chemical conditions of today, provided the terrestrial carbon cycle remains a carbon sink until the end of the 21st century. Given the terrestrial carbon cycle would turn into a carbon source as suggested by a few model simulations^34^ provided by the Coupled Climate-Carbon Cycle Model Intercomparison Project (C${}^{4}$MIP), an early deployment of atmospheric CDR measures would be much less effective (Supplementary Figs. 1 and 2 generated with modified parameters for the terrestrial submodel, see Supplementary Table 1). The latter indicates that the future development of the terrestrial biosphere is deeply connected with the marine biosphere and has the potential to accelerate the decrease of biodiversity in the ocean. Accompanying aggressive climate mitigation pathways with CDR deployment at an early stage could help to dampen the most severe impacts on key marine ecosystems. To have a significant effect, though, it is crucial that CDR technologies are deployed as early as possible, ideally within the next decade.

## Methods

### CLIMBER-3$\alpha$ + C

The following describes the Earth system model employed and the experimental design of the simulations conducted. CLIMBER-3$\alpha$ + C is based on the EMIC^51^ CLIMBER-3$\alpha$ which was described by Montoya et al.^31^ in detail. Since then, the model has been revised and extended with respect to its physical and biogeochemical components. In the following section, we present the most important innovations/modifications added to the original code.

### Atmosphere

As its precursor, CLIMBER-3$$\alpha$$ + C still employs the statistic dynamical atmosphere POTSDAM-2 (Potsdam Statistical Dynamical Atmospheric Model 2)^52^, with a horizontal resolution of 7.5$${}^{\circ }$$$$\times$$ 22.5$${}^{\circ }$$. The model realistically reproduces large scale structures, such as the Hadley circulation and the main high- and low-pressure systems, but does not resolve synoptic scale processes. The assumption of universal vertical structures of temperature and humidity allows for the reduction in their dynamic equations to a two-dimensional problem. The atmospheric wind velocities are provided at 10 different pressure levels. When calculating the longwave radiative transport, a 16 level scheme is employed^31^. The vegetation cover is prescribed as it is in the original version of CLIMBER-3$$\alpha$$.

Because aeolian dust is one of the most important iron sources for marine biota, we have implemented an atmospheric dust transport model following Bauer and Ganopolski^53^. Similar to atmospheric humidity, it assumes an exponential vertical profile for the dust mixing ratios $M(z)$:

1 $$M(z)={M}_{{\rm{s}}}\cdot exp(-z/{h}_{{\rm{d}}}),$$

with ${h}_{{\rm{d}}}$ representing the scale hight of the dust mixing ratio (2000 m). ${M}_{{\rm{s}}}$ represents the near surface dust mixing ratio. If

2 $$\rho (z)={\rho }_{{\rm{s}}}\cdot exp(-z/{h}_{{\rm{a}}})$$

is the vertical profile of air density, with the atmospheric scale of hight ${h}_{{\rm{a}}}$ and near-surface density of ${\rho }_{{\rm{s}}}$, then the vertically integrated dust concentration is given by:

3 $$D=\int_{{z}_{{\rm{s}}}}^{{H}_{{\rm{a}}}} M\rho \,dz$$

and the balance equation, which includes advective and diffusive transports, sources and sinks, reads:

4 $$\frac{\partial D}{\partial t}=-{\nabla }_{{\rm{H}}}\left(\int_{z_{{\rm{s}}}}^{H_{{\rm{a}}}}\rho \overrightarrow{u}M\,dz- \int_{{z}_{{\rm{s}}}}^{{H}_{{\rm{a}}}}\rho K{\nabla }_{{\rm{H}}}M\,dz\right)+Q-{R}_{{\rm{d}}}-{R}_{{\rm{w}}},$$

where

5 $${\nabla }_{{\rm{H}}}=\overrightarrow{i}\frac{\partial }{\partial x}+\overrightarrow{j}\frac{\partial }{\partial y}$$

and $\overrightarrow{u}$ represents the wind velocity vector. $Q$ represents the dust emission flux, which depends on the land surface type, erosion and the wind-dependent uplift of dust into the atmosphere^53^. ${R}_{{\rm{d}}}$ and ${R}_{{\rm{w}}}$ represent the dry and wet dust deposition rates, respectively. The parameters were taken from Bauer and Ganopolski^53^.

### Ocean general circulation model

CLIMBER-3$\alpha$ + C utilizes GFDL’s three dimensional $z$-coordinate Modular Ocean Model (version 3.1) (MOM-3.1)^54^ with the upgrades described in Hofmann and Maqueda^55^. This version benefits from the improved accuracy of a second-order moments advection scheme^56^ which strictly limits spurious numerical diffusion and dispersion and employs a realistically low diapycnal background diffusivity of 10${}^{-5}$ m${}^{2}$s${}^{-1}$. Furthermore, the model accounts for the effects of geothermal heating^57^ and a circulation dependent parameterization of the Gent-McWilliams diffusivity^58,59^ varying between 275 and 1100 m${}^{2}$s${}^{-1}$ in space and time. The models horizontal resolution is 3.75${}^{\circ }\times$3.75${}^{\circ }$ while the vertical dimension is divided in 24 layers with thickness increasing with depth from 25 m at the top to a maximum of 514 m at the bottom. Save for the wind-stress fields, which are externally provided from a monthly mean climatology^60^, the OGCM exchanges radiative-, heat- and buoyancy fluxes with the atmosphere (POTSDAM-2) and the sea-ice model (ISIS)^61^. After a spin-up for more than 6000 years the model well reproduces the large scale pattern of temperature and salinity while the maximum strength of the Atlantic meridional overturning circulation (AMOC) stabilizes at 14.4 Sv (1 Sv = 10${}^{6}$ m${}^{3}$ s${}^{-1}$).

### Terrestrial box model

**Fig. 7:**
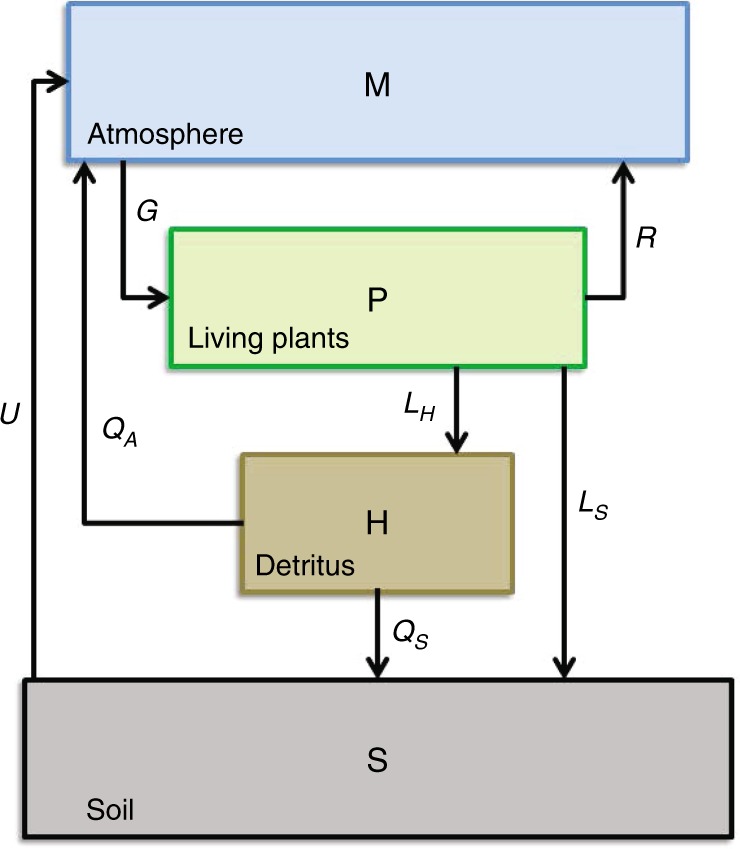
Terrestrial carbon cycle model. Box model of the terrestrial carbon cycle comprising the carbon pools and its exchange fluxes for the atmosphe (M), the living plants (P), the detritus (H) and the soil (S) (redrawn after Wigley^33^).

CLIMBER-3$\alpha$ + C does not include a model of the terrestrial biosphere. To, at least, emulate the response of the terrestrial carbon cycle to variations in atmospheric temperature and CO${}_{2}$ concentration, a box model similar to that described by Wigley^33^ was coupled with the atmospheric sub-model of CLIMBER-3$\alpha$ + C. The box-model comprises four boxes: (1) atmosphere ($M$), (2) living plants ($P$), (3) detritus ($H$) and (4) soil ($S$) (see Fig. 7), which can exchange carbon fluxes among each other governed by the following equations.

6 $$\frac{dM}{dt}=I-F-(G-R-{Q}_{A}-U)$$

7 $$\frac{dP}{dt}={G}_{P}-R-L$$

8 $$\frac{dH}{dt}={G}_{H}+{L}_{H}-Q$$

9 $$\frac{dS}{dt}={G}_{S}+{Q}_{S}+{L}_{S}-U$$

Here $I$ represents the anthropogenic net emission rate of CO${}_{2}$, and $F$ represents the flux from the atmosphere to the ocean. The gross primary production $G$ is equal to the sum of ${G}_{P}={g}_{P}G$, ${G}_{H}={g}_{L}G$, and ${G}_{S}=(1-{g}_{P}-{g}_{L})G$: $G={G}_{P}+{G}_{H}+{G}_{S}$ and $Q$ represents the sum of the remineralization fluxes from detritus to the atmosphere (${Q}_{A}$) and soil reservoir (${Q}_{S}$): $Q={Q}_{A}+{Q}_{S}$. $R$ represents the respiration flux from living plants into the atmosphere, and $U$ represents the outgassing of soil due to remineralization. The carbon flux $L$ from dying plants can be written as the sum of the fluxes into detritus and the soil reservoir: ${L}_{H}+{L}_{S}$.

Following Wigley^33^ the fluxes $$L$$, $$Q$$ and $$U$$ can be related to specific decay time constants $$\tau$$: $$L={q}^{P}$$ $$\cdot$$ $$P/{\tau }_{P}$$, $$Q={q}^{HS}$$ $$\cdot$$ $$H/{\tau }_{H}$$, and $$U={q}^{HS}$$ $$\cdot$$ $$S/{\tau }_{S}$$, while $${G}_{P}={r}_{{\rm{m}}}$$ $$\cdot$$ $${g}_{P}$$ $$\cdot$$ $${G}_{0}$$, $$R={r}_{{\rm{m}}}$$ $$\cdot$$ $${R}_{0}$$, $${G}_{H}={r}_{{\rm{m}}}$$ $$\cdot$$ $${g}_{L}$$ $$\cdot$$ $${G}_{0}$$, $${L}_{H}=\Phi$$ $$\cdot$$ $$L$$, $${Q}_{A}$$ = $$(1-\lambda)$$ $$\cdot$$ $$Q$$, $${G}_{S}={r}_{{\rm{m}}}$$ $$\cdot$$ $$(1-{g}_{P}-{g}_{L})$$ $$\cdot$$ $${G}_{0}$$, $${Q}_{S}$$ = $$\lambda$$$$\cdot Q$$, and $${L}_{S}$$ = $$(1-\Phi)$$ $$\cdot$$ $$L$$.

The temperature dependent quantities ${q}^{P}$ and ${q}^{HS}$ are given by:

10 $${q}^{P}={({Q}_{10}^{P})}^{({T}_{{\rm{ATM}}}/10)}$$

11 $${q}^{HS}={({Q}_{10}^{HS})}^{({T}_{{\rm{ATM}}}/10)},$$

with the ${Q}_{10}^{P}$ and ${Q}_{10}^{HS}$ factors given in Table 1 and ${T}_{{\rm{ATM}}}$ representing the globally avaraged surface air temperature.

**Table 1:** Parameters for the terrestrial carbon cycle box model.

Parameter	Value default	Unit
*G*${}_{0}$	76	GtC year${}^{-1}$
*R*${}_{0}$	14	GtC year${}^{-1}$
*Q*${}_{10}^{P}$	1.4	—
*Q*${}_{10}^{HS}$	1.4	—
${\tau }_{P}$	60	years
${\tau }_{H}$	3	years
${\tau }_{S}$	400	years
$\Phi$	0.98	—
*g*${}_{P}$	0.35	—
*g*${}_{L}$	0.6	—
*r*	1.4	—
$\lambda$	0.05	—

Given, $N$ represents the net primary production (NPP) at a given time and ${N}_{0}$ represents the preindustrial NPP then^62^:

12 $$\frac{N}{{N}_{0}}=\frac{(C-{C}_{b})(1+b({C}_{0}-{C}_{b}))}{({C}_{0}-{C}_{b})(1+b(C-{C}_{b}))}={r}_{{\rm{m}}},$$

where $C$ is the atmospheric CO${}_{2}$ concentration, ${C}_{0}$ = 280 ppm, and ${C}_{b}$ = 31 ppm and

13 $$b=\frac{(680-{C}_{b})-r(340-{C}_{b})}{(r-1)(680-{C}_{b})(340-{C}_{b})},$$

The value for $r$ is given in Table 1.

To find a parameter set for emulating the terrestrial biosphere under anthropogenic emissions during the late 21st century, we conducted several test runs, where we coupled the terrestrial box model with a simple box model of the ocean carbon cycle. Therefore, we were able to quickly run a large number of experiments with different model parameters. The final parameter set derived from these experiments is listed in Table 1.

### Ocean carbon cycle and marine ecosystem models

The ocean carbon cycle and marine ecosystem model mainly bases on an upgraded version of the model by Six and Maier-Reimer^63^ and prognostically solves for the spatio-temporal evolution of dissolved inorganic carbon (DIC), total alkalinity, phosphate, oxygen, silicate, phytoplankton, zooplankton, dissolved organic carbon (DOC), particulate organic carbon (POC), calcite, and dissolved iron. It assumes throughout a constant stoichiometric relationship between carbon (C), nitrate (NO${}_{3}^{-}$), phosphate (PO${}_{4}^{-}$), and oxygen (O${}_{2}$) of organic matter (Redfield-ratio^63,64^): C : NO${}_{3}^{-}$ : H${}_{2}$PO${}_{4}^{-}$ : O${}_{2}$ = 122 : 16 : 1 : (−172)

The model is initialized with a homogeneous distribution of DIC (2341 $\upmu {\mathrm{molL}}^{-1}$), total alkalinity (2503 $\upmu {\mathrm{equL}}^{-1}$), inorganic phosphate (2.3 $\upmu {\mathrm{molL}}^{-1}$) and silicate (80.0 $\upmu {\mathrm{molL}}^{-1}$). We utilize the acidity constants given by Zeebe and Wolf-Gladrow (2002)^65^. Air-sea gas-exchange of CO${}_{2}$ is parameterized according to Wanninkhof^66^ as a quadratic function of the wind speed while oxygen fluxes are calculated from a simple restoring of sea surface concentrations to thermodynamically equilibrium values^67^. CLIMBER-3$\alpha$ + C accounts for the dynamics of dissolved iron by assuming only aeolian dust as the only source and including its co-limitation effects on the phytoplankton growth rates^68^ as well as the mineral ballast effect^69,70^.

### Experimental design

The base “SSP1-2.6” emission scenario starting in 2005 was linearly extended from year 2100 towards year 2150, where net emission rates were assumed to approach zero. To construct the emission scenario “EARLY”, we have subtracted annual CO${}_{2}$ emission rates *f*(t) from “SSP1-2.6” by imposing a logistic function between 2025 and 2050, where t represents the number of years.

14 $$f(t)=\frac{L}{1+exp(-nLt)(L-1)}$$

With $L$ = 4.750 and $n$ = 0.2, CO${}_{2}$ emission rates are assumed to drop to zero by year 2050. From here on, we assume zero net anthropogenic emission rates until the end of our simulations in year 2200. Within the first 25 years until the peak of the additional CDR is reached (2025–2050), an extra amount of 109 GtC is removed from the atmosphere. From 2050 until 2075 (i.e., until the time when the “SSP1-2.6” scenario meets the zero emissions level), the “EARLY” scenario removes a further total of 59 GtC from the “SSP1-2.6” scenario which keeps net emissions at the zero line.

After 2075, the “SSP1-2.6”-emissions scenario reached net negative values on the order of −2.5 GtC yr${}^{-1}$ by the end of the 21st century. As a result, “SSP1-2.6” requires total net negative emissions of 105 GtC between 2075 and 2150, while during the same period, “EARLY” assumes net zero emissions.

In constructing “LATE”, we used the same procedure based on the above logistic function. However, we applied the extra CDR measures 50 years later than those in the case of “EARLY”, starting in year 2075 and lasting until 2100.

To ensure the same additional cumulative carbon removal of 63 GtC relative to “SSP1-2.6” in both the “EARLY” and “LATE” scenarios over the whole simulation time, we included a period of only mild net negative emissions after 2100 in the “LATE” scenario, which linearly decreased to zero in 2150.

### Gross Calcification rates of coral reefs

Silverman et al.^41^ have determined the calcification rates for more than 9000 reef location. They utilized a previosly found correlation between rates of inorganic aragonite precipitation ${G}_{{\rm{i}}}$

15 $${G}_{{\rm{i}}}=\frac{24}{1000}\cdot \left(-0.0177\cdot {T}^{2}+1.4697\cdot T+14.893\right)\cdot {\left({\Omega }_{{\rm{arg}}}-1\right)}^{\left(0.0628\cdot T+0.0985\right)}$$

and the temperature dependent gross calcification rates ${}^{T}{G}_{{\rm{gross}}}$

16 $${}^{T}{G}_{{\rm{gross}}}={A}_{{\rm{c}}}\cdot {k}_{{\rm{r}}}^{^{\prime} }\cdot {G}_{{\rm{i}}}\cdot exp\left(-{\left(\frac{k^{\prime}_{p} \cdot (T-{T}_{{\rm{opt}}})}{{\Omega }_{{\rm{a}}}^{2}}\right)}^{2}\right),$$

where ${A}_{{\rm{c}}}$ and ${k}_{{\rm{r}}}^{{\prime} }$ represent the relative life coral cover of a reef (between 0 and 1) and a specific area constant, respectively (for more details, see Silverman et al.^41^).

## Supplementary information

Supplementary information for: Strong time dependence of ocean acidification mitigation by atmospheric carbon dioxide removal. by M. Hofmann et al.

## Data Availability

The data generated by our model simulations are available from the authors on request.

## Code availability

The CLIMBER-3α + C code employed in this study is available from the authors upon request.

## References

[1] IPCC2013. *Climate Change 2013: The Physical Science Basis. Contribution of Working Group I to the Fifth Assessment Report of the Intergovernmental Panel on Climate Change* (Cambridge University Press, Cambridge, United Kingdom and New York, NY, USA, 2013). www.climatechange2013.org.

[2] Caldeira K, Wickett ME (2003). Oceanography: anthropogenic carbon and ocean pH. Nature.

[3] Schellnhuber H, Rahmstorf S, Winkelmann R (2016). Why the right climate target was agreed in Paris. Nat. Clim. Change.

[4] Steffen W (2018). Trajectories of the Earth system in the anthropocene. Proc. Natl Acad. Sci. USA.

[5] IPCC2018. In *Global Warming of 1.5*$${}^{\circ }$$*C an IPCC special report on the impacts of global warming of 1.5*$${}^{\circ }$$*C above pre-industrial levels and related global greenhouse gas emission pathways, in the context of strengthening the global response to the threat of climate change, sustainable development, and efforts to eradicate poverty* (http://ipcc.ch/report/sr15/).

[6] Rogelj J (2016). Paris agreement climate proposals need a boost to keep warming well below 2$${}^{\circ}$$ C. Nature.

[7] Mathesius S, Hofmann M, Caldeira K, Schellnhuber HJ (2015). Long-term response of oceans to CO$${}_{2}$$ removal from the atmosphere. Nat. Clim. Change.

[8] Magnan AK (2016). Implications of the Paris agreement for the ocean. Nat. Clim. Change.

[9] Le Quéré C (2012). The global carbon budget 1959-2011. Earth Syst. Sci. Data.

[10] Hoegh-Guldberg (2007). Coral reefs under rapid climate change and ocean acidification. Science.

[11] Hofmann M, Schellnhuber HJ (2010). Ocean acidification: a millennial challenge. Energy Environ. Sci..

[12] Frieler K (2012). Limiting global warming to 2$${}^{\circ }$$ C is unlikely to save most coral reefs. Nat. Clim. Change.

[13] Gardner J, Manno C, Bakker DCE, Peck VL, Geraint Tarling GA (2018). Southern ocean pteropods at risk from ocean warming and acidification. Mar. Biol..

[14] Hoegh-Guldberg O, Kennedy EV, Beyer HL, Mcclennen C, Possingham HP (2018). Securing a long-term future for coral reefs. Trends Ecol. Evol..

[15] Schleussner C-F (2016). Differential climate impacts for policy-relevant limits to global warming : the case of 1 . 5 C and 2 C. Earth Syst. Dynam..

[16] Anthony KRN (2016). Coral reefs under climate change and ocean acidification: challenges and opportunities for management and policy. Annu. Rev. Environ. Resour..

[17] McNeil BI, Matear RJ (2008). Southern ocean acidification: a tipping point at 450-ppm atmospheric CO$${}_{2}$$. Proc. Natl Acad. Sci. USA.

[18] Bednaršek N, Harvey CJ, Kaplan IC, Feely RA, Mozina J (2016). Pteropods on the edge: cumulative effects of ocean acidification, warming, and deoxygenation. Prog. Oceanogr..

[19] Vaughan NE, Lenton TM, Shepherd JG (2009). Climate change mitigation: trade-offs between delay and strength of action required. Clim Change.

[20] Frölicher TL, Joos F (2010). Reversible and irreversible impacts of greenhouse gas emissions in multi-century projections with the NCAR global coupled carbon cycle-climate model. Clim. Dyn..

[21] Schellnhuber HJ (2011). The good, the MAD, and the sensible. Proc. Natl Acad. Sci. USA.

[22] Caldeira K, Bala G, Cao L (2013). The science of geoengineering. Annu. Rev. Earth Planet. Sci..

[23] Cao L, Caldeira K (2010). Atmospheric carbon dioxide removal: Long-term consequences and commitment. Environ. Res. Lett..

[24] Vaughan NE, Lenton TM (2009). A review of climate geoengineering proposals. Clim. Change.

[25] *Reflecting Sunlight to Cool Earth*. (The National Academies Press, National Research Council Climate Intervention, 2015).

[26] Lackner KS (2003). A guide to CO$${}_{2}$$ sequestration. Science.

[27] Bauer N (2017). Shared socio-economic pathways of the energy sector – quantifying the narratives. Global Environ. Change.

[28] Kriegler E, Edenhofer O, Reuster L, Luderer G, Klein D (2013). Is atmospheric carbon dioxide removal a game changer for climate change mitigation?. Clim. Change.

[29] van Vuuren PD (2017). Energy, land-use and greenhouse gas emissions trajectories under a green growth paradigm. Global Environ. Change.

[30] Smith P (2016). Biophysical and economic limits to negative CO2 emissions. Nat. Clim. Change.

[31] Montoya M (2005). The earth system model of intermediate complexity CLIMBER-3. Part I: description and performance for present-day conditions. Clim. Dyn..

[32] Boucher O (2012). Reversibility in an Earth System model in response to CO$${}_{2}$$ concentration changes. Environ. Res. Lett..

[33] Wigley TML (1993). Balancing the carbon budget. Implications for projections of future carbon dioxide concentration changes. Tellus.

[34] Friedlingstein P (2006). Climate-carbon cycle feedback analysis: results from the C$${}^{4}$$ MIP model intercomparison. J. Clim..

[35] Meinshausen M (2011). The RCP greenhouse gas concentrations and their extension from 1765 to 2300. Clim. Change.

[36] IPCC2014. In Field, C. et al. (eds.) *Climate Change 2014: Impacts, Adaptation, and Vulnerability. Part A: Global and Sectoral Aspects. Contribution of Working Group II to the Fifth Assessment Report of the Intergovernmental Panel on Climate Change* (Cambridge University Press, Cambridge, United Kingdom and New York, NY, USA).

[37] Johan Rockström J (2017). A roadmap for rapid decarbonization. Science.

[38] Rogelj J (2018). Scenarios towards limiting global mean temperature increase below 1.5 $${}^{\circ }$$C. Nat. Clim. Change.

[39] Sabine CL (2003). The oceanic sink for anthropogenic CO$${}_{2}$$. Science.

[40] Hauri C, Friedrich T, Timmermann A (2016). Abrupt onset and prolongation of aragonite undersaturation events in the Southern ocean. Nat. Clim. Change.

[41] Silverman J, Lazar B, Cao L, Caldeira K, Erez J (2009). Coral reefs may start dissolving when atmospheric CO$${}_{2}$$ doubles. Geophys. Res. Lett..

[42] Mollica NR (2017). Ocean acidification affects coral growth by reducing skeletal density. Proc. Natl Acad. Sci. USA.

[43] David C (2016). Community structure of under-ice fauna in relation to winter sea- ice habitat properties from the Weddell Sea. Polar Biol..

[44] Feely RA (2016). Estuarine, Coastal and Shelf Science Chemical and biological impacts of ocean acidification along the west coast of North America. Estuar. Coast. Shelf Sci..

[45] Langdon, C. & Atkinson, M. J. Effect of elevated pCO$${}_{2}$$ on photosynthesis and calcification of corals and interactions with seasonal change in temperature/irradiance and nutrient enrichment. *J. Geophys. Res.* **110**, C09S07 10.1029/2004JC002576 (2005).

[46] Guinotte JM, Buddemeier RW, Kleypas JA (2003). Future coral reef habitat marginality: temporal and spatial effects of climate change in the Pacific basin. Coral Reefs.

[47] Cyronak T (2018). Taking the metabolic pulse of the world’s coral reefs. PloS ONE.

[48] Fabricius KE (2011). Losers and winners in coral reefs acclimatized to elevated carbon dioxide concentrations. Nat. Clim. Change.

[49] Hughes TP (2017). Coral reefs in the Anthropocene. Nature.

[50] Hughes TP (2018). Global warming transforms coral reef assemblages. Nature.

[51] Claussen M (2002). Earth system models of intermediate complexity: closing the gap in the spectrum of climate system models. Clim. Dyn..

[52] Petoukhov V (2000). CLIMBER-2: a climate system model of intermediate complexity. Part I: model description and performance for present climate. Clim. Dyn..

[53] Bauer E, Ganopolski A (2010). Aeolian dust modeling over the past four glacial cycles with CLIMBER-2. Global Planet. Change.

[54] Pacanowski, R. C. & Griffies, S. M. The MOM-3 Manual. Tech. Rep. 4, GFDL Ocean Group (NOAA/Geophysical Fluid Dynamics Laboratory, Princeton, NJ, 1999).

[55] Hofmann M, MoralesMaqueda MA (2006). Performance of a second-order moments advection scheme in an Ocean General Circulation Model. J. Geophys. Res..

[56] Prather MJ (1986). Numerical advection by conservation of second-order moments. J. Geophys. Res..

[57] Hofmann, M. & Maqueda, M. A. M. Geothermal heat flux and its influence on the oceanic abyssal circulation and radiocarbon distribution. *Geophys. Res. Lett.* **36**, 6671–6681 (2009).

[58] Gent PR, McWilliams JC (1990). Isopycnal mixing in ocean circulation models. J. Phys. Oceanogr..

[59] Hofmann M., Morales Maqueda M. A. (2011). The response of Southern Ocean eddies to increased midlatitude westerlies: A non-eddy resolving model study. Geophysical Research Letters.

[60] Trenberth, K., Olson, J. & Large, W. A global ocean wind stress climatology based on ECMWF analyses. 10.5065/D6ST7MR9 (1989).

[61] Fichefet T, Maqueda MAM (1997). Sensitivity of a global sea ice model to the treatment of ice thermodynamics and dynamics. J. Geophys. Res..

[62] Meinshausen M, Raper CB, Wigley TML (2011). Emulating coupled atmosphere-ocean and carbon cycle models with a simpler model, MAGICC6 – Part 1: Model description and calibration. Atmos. Chem. Phys..

[63] Six KD, Maier-Reimer E (1996). Effects of plankton dynamics on seasonal carbon fluxes in an ocean general circulation model. Global Biogeochem. Cycles.

[64] Takahashi T, Broecker WS, Langer S (1985). Redfield ratio based on chemical data from isopycnal surfaces. J. Geophys. Res..

[65] Zeebe R, Wolf-Gladrow DA (2001). CO2 in Seawater: Equilibrium, Kinetics, Isotopes.

[66] Wanninkhof R (1992). Relationship between wind speed and gas exchange over the ocean. J. Geophys. Res..

[67] Garcia HE, Gordon LI (1992). Oxygen solubility in seawater: Better fitting equations. Limnol. Oceanogr..

[68] Parekh P., Follows M. J., Boyle E. A. (2005). Decoupling of iron and phosphate in the global ocean. Global Biogeochemical Cycles.

[69] Klaas C, Archer DE (2002). Association of sinking organic matter with various types of mineral ballast in the deep sea: implications for the rain ratio. Global Biogeochem. Cycles.

[70] Hofmann M, Schellnhuber H-J (2009). Oceanic acidification affects marine carbon pump and triggers extended marine oxygen holes. Proc. Natl Acad. Sci. USA.

