# Supplementary material for: Strong time dependence of ocean acidification mitigation by atmospheric carbon dioxide removal
Source: Nat Commun. 2019 Dec 6;10:5592. doi: 10.1038/s41467-019-13586-4 (PMC6898155; doi:10.1038/s41467-019-13586-4)
Supplement: Supplementary file 1 — Supplementary information for: Strong time dependence of ocean acidification mitigation by atmospheric carbon dioxide removal. by M. Hofmann et al. [file 41467_2019_13586_MOESM1_ESM.pdf]

# Supplementary information for: Strong time dependence of ocean acidification mitigation by atmospheric carbon dioxide removal.

**by M. Hofmann et al.**

**Supplementary Note: Sensitivity of presented results on terrestrial carbon cycel box model parameters**

By investigating the response of 11 models comprising the Coupled ClimateCarbon Cycle Model Intercomparison Project ($C^4$MIP) Friedlingstein et al. (2006) [1] have shown that the simulated carbon uptake of the land biosphere varies between about -6 and +11 GtC per year by 2100. While the majority of the models simulates a carbon sink under anthropogenic $CO_2$ emissions three of them turn from a sink into a source of $CO_2$ in the second half of the twenty-first century. Notably the terrestrial carbon cycle component of the HadCM3LC model turns into a significant carbon source of about 6 GtC in year 2100.

Because of this enormous uncertainty in simulating land to atmosphere fluxes of $CO_2$ we have become motivated to perform a second suite of model runs with a parameter set where the terrestrial box model version[2] was tuned such that the land biosphere remains a net carbon sink only until approximately 2050 but turns into a net carbon source, afterwards (Supplementary Figure 1) . The modified parameters for the terrestrial carbon cycle box model are listed in table 1.

Utilizing the modified parameter set for the terrestrial box model (Supplementary Table 1), neither EARLY nor LATE will lead to a satisfying preservation of calcification rates in coral reefs against the SSP1-2.6 scenario as reported in the standard run of our paper (Supplementary Figure 2).

Supplementary Table 1: Parameters for second version of the terrestrial carbon cycle box model revealing a carbon source during the second half of the twenty-first century.

| Parameter | Value default | Unit |
|---|---|---|
| $G_0$ | 380 | GtC $year^{-1}$ |
| $R_0$ | 70 | GtC $year^{-1}$ |
| $Q_{10}^{P}$ | 3.5 | - |
| $Q_{10}^{HS}$ | 3.5 | - |
| $\tau_P$ | 60 | years |
| $\tau_H$ | 3 | years |
| $\tau_S$ | 400 | years |
| $\Phi$ | 0.98 | - |
| $g_P$ | 0.35 | - |
| $g_L$ | 0.6 | - |
| r | 1.4 | - |
| $\lambda$ | 0.05 | - |

## Supplementary Figures

**Supplementary Figure 1** Time evolution of atmospheric and global mean sea surface values of climatologically relevant parameters during the entire model simulation between years 1800 and 2200 employing the modified parameter set for terrestrial carbon cycle (see Supplementary Table 1). a) atmospheric $pCO_2$ concentrations in ppmv; b) globally averaged and annual mean atmospheric temperature anomaly at the Earth surface in $^\circ$C; c) sea surface pH anomaly; d) uptake of $CO_2$ by the ocean in GtC per year; d) uptake of $CO_2$ by the land biosphere in GtC per year.The light blue and the pink stripe mark the period of the initial CDR measures for the EARLY and LATE scenarios, respectively.

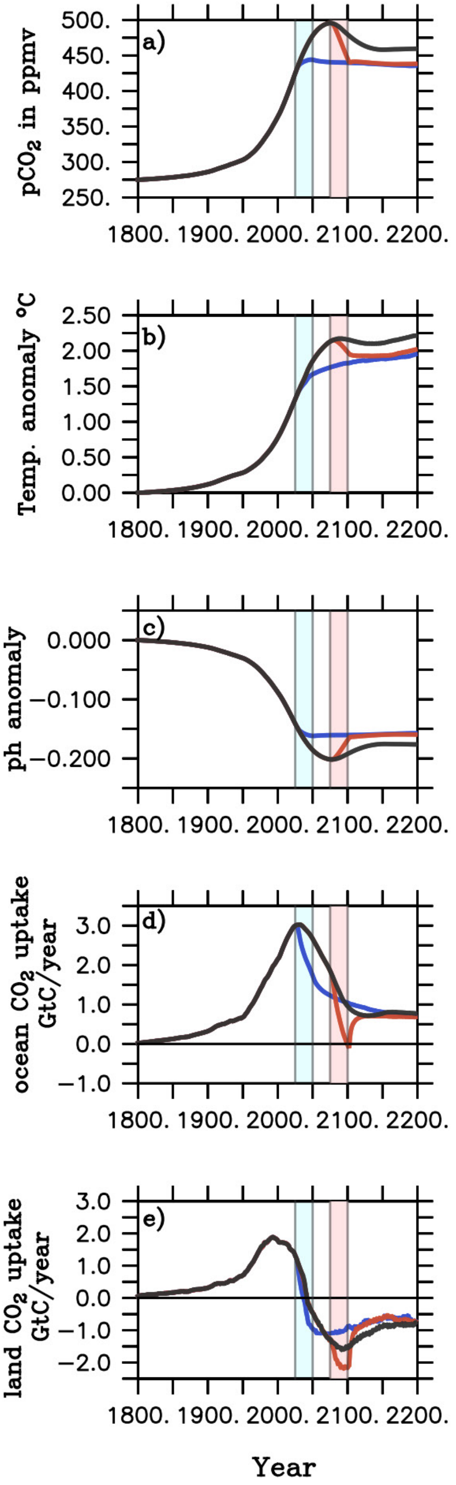
pCO2 in ppmv
a)
500.
450.
400.
350.
300.
250.
1800. 1900. 2000. 2100. 2200.
Temp. anomaly °C
b)
2.50
2.00
1.50
1.00
0.50
0.00
1800. 1900. 2000. 2100. 2200.
ph anomaly
c)
0.000
−0.100
−0.200
1800. 1900. 2000. 2100. 2200.
ocean CO2 uptake
GtC/year
d)
3.0
2.0
1.0
0.0
−1.0
1800. 1900. 2000. 2100. 2200.
land CO2 uptake
GtC/year
e)
3.0
2.0
1.0
0.0
−1.0
−2.0
1800. 1900. 2000. 2100. 2200.
Year

**Supplementary Figure 2** Global distribution of tropical coral reefs from the Reef Base (M. Tupper et al., ReefBase: A Global Information System on Coral Reefs, http://www.reefbase.org) and their corresponding decadal mean gross calcification rates ${}^{T}G_{\mathrm{gross}}$ relative to preindustrial values in %: (2040 - 2050) a) EARLY; c) LATE; e) SSP1-2.6; (2070 - 2080) b) EARLY; d) LATE; f) SSP1-2.6;

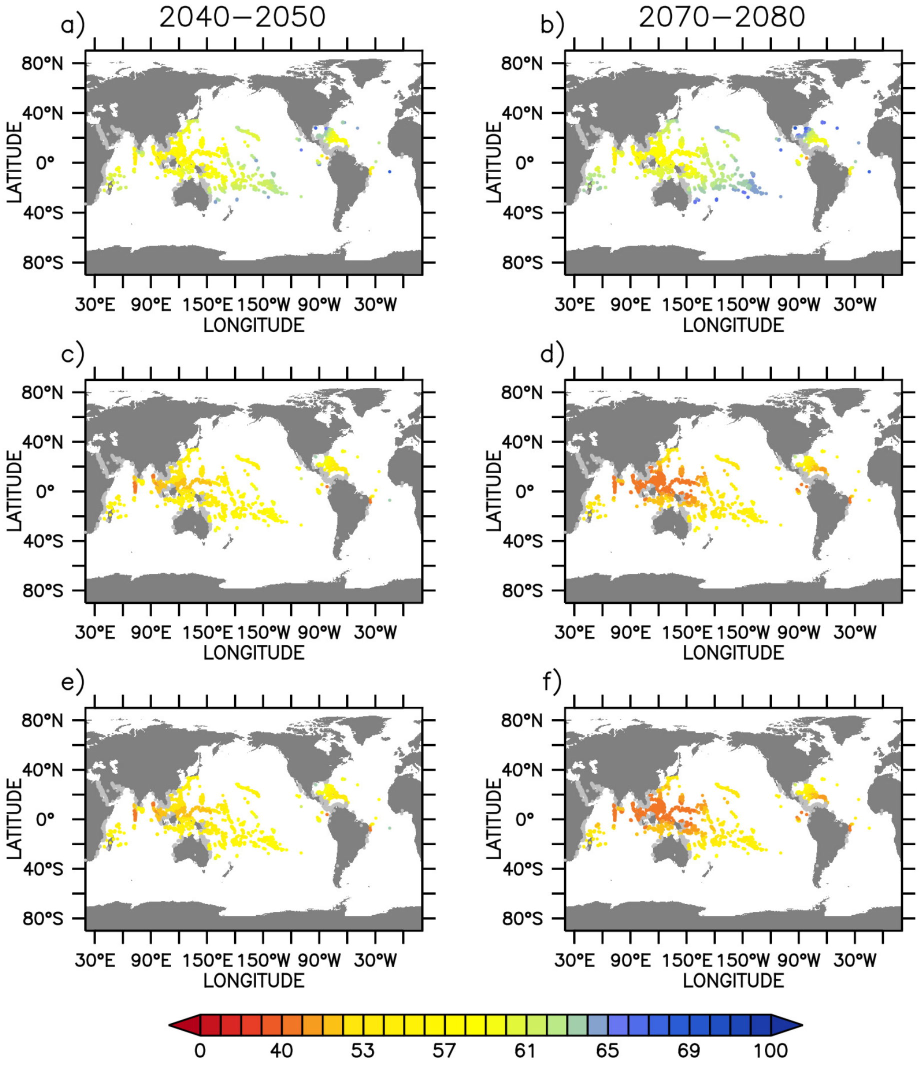

## Supplementary References


1. Friedlingstein, P. *et al.* Climate-Carbon Cycle Feedback Analysis: Results from the $C^4$MIP Model Intercomparison. *Journal of Climate* **19**, 3337–3353 (2006).

2. Wigley, T. M. L. Balancing the carbon budget. Implications for projections of future carbon dioxide concentration changes. *Tellus B: Chemical and Physical Meteorology* **45**, 409–425 (1993).
